# Focusing of mid-infrared polaritons through patterned graphene on van der Waals crystals

**DOI:** 10.1515/nanoph-2023-0778

**Published:** 2024-04-15

**Authors:** Ruey-Tarng Liu, Yan-Ze Wu, Chia-Chien Huang

**Affiliations:** Department of Physics and Graduate Institute of Nanoscience, 34916 National Chung Hsing University, 145 Xingda Rd., Taichung, 40227, Taiwan

**Keywords:** focusing, graphene, mid-infrared, hyperbolic polaritons, van der Waals crystals

## Abstract

Manipulating the propagation of mid-infrared (mid-IR) light is crucial for optical imaging, biosensing, photocatalysis, and guiding photonic circuits. Artificially engineered metamaterials were introduced to comprehensively control optical waves. However, fabrication challenges and optical losses have impeded the progress. Fortunately, two-dimensional van der Waals (vdW) materials are alternatives because of their inherent optical properties, such as hyperbolic behavior, high confinement, low loss, and atomic-scale thickness. In this research, we conducted theoretical and numerical investigations on the *α*-phase molybdenum trioxide, a biaxial vdW material, with patterned graphene to assess the potential of the tunable focusing of mid-IR light. Our proposed method directly alters the path of excited light to focus mid-IR light by negative refraction. Further, the patterned graphene in our design offers enhanced focusing characteristics, featuring a significantly reduced waist diameter with 1/92 of the free-space wavelength, an enhanced beam quality without pronounced field ripples, and a fivefold increase in field intensity. Moreover, our approach significantly preserves the waist diameter of the focused beam while facilitating directional steering. Thus, the focused beam can propagate in a canalized manner toward the desired direction. These advancements lay the foundation for promising applications in planar photonics.

## 1 Introduction

Focusing electromagnetic waves at deep subwavelength scales has garnered substantial attention across a wide spectral range, encompassing ultraviolet (UV) [1], [2] to infrared (IR) [3], [4] regions. This exploration has various applications, including light–matter interaction enhancement [5], [6], light confinement [7], [8], imaging resolution improvements [9], [10], chemical detection [11], [12], and biosensing [13], [14]. Traditionally, metamaterials (MMs) [15], composite materials often incorporating metals, are employed for light focusing, particularly in the UV to near-IR spectral domains [16], by constructing hyperbolic media with indefinite wavevectors. However, MMs exhibit limitations in their responses within the mid-IR and terahertz frequency bands [17]. Furthermore, the fabrication of hyperbolic media using bulk materials presents notable challenges. Natural two-dimensional (2D) van der Waals (vdW) layered materials [18], [19], [20], such as graphene [21], [22], hexagonal boron nitride (hBN) [23], *α*-phase molybdenum trioxide (*α*-MoO_3_) [24], and transition metal dichalcogenides [25], are suitable for addressing these limitations. These materials can support diverse types of polaritons, offering distinct advantages, such as low-loss properties [26], giant optical anisotropy [27], and ultrahigh mode confinement [14], [28], [29]. Thus, they are suitable for mid-IR light focusing.

Polaritons [30], [31], quasi-particles resulting from the hybridization of light–matter interactions, play a crucial role in photonics; they include plasmon polaritons, phonon polaritons (PhPs), and exciton polaritons. The PhPs exhibit distinctive advantages, characterized by their low-loss properties and highly confined fields. However, challenges arise when considering natural hBN crystals, which inherently possess out-of-plane hyperbolicity [32], [33]. This inherent property impedes the development of photonic devices compatible with on-chip planar fabrication processes. Recently, a natural *α*-MoO_3_ crystal, exhibiting in-plane hyperbolicity within the mid-IR and terahertz frequency ranges [34], [35], has been identified. This unique crystal sustains in-plane hyperbolic PhPs (HPhPs) and offers a versatile platform for wavefront control, directional canalization, negative refraction, and focusing. These capabilities are harnessed by manipulating crystal interlayer orientations [36], [37], [38] and interfacing [39], [40] *α*-MoO_3_ with various vdW crystals.

Recent studies have investigated the subwavelength focusing of in-plane HPhPs, utilizing plasmonic antennas within hBN [41] and *α*-MoO_3_ slabs [42], [43], [44], [45], [46]. For the hBN system, a metallic disk positioned beneath the crystal is employed to launch and concentrate the polaritons along the vertical axis relative to the hBN slab, presenting challenges in its integration with planar optical chips [41]. Contrarily, a convex metallic antenna is engineered on the *α*-MoO_3_ slab, which is effective for exciting and focusing the polaritons within the plane of the *α*-MoO_3_. The tuning of the focusing characteristics primarily hinges on the control of the geometric parameters of the metallic antenna [42], [43], [44]. The authors in Ref. [45] achieved bending-free refraction of HPhPs in *α*-MoO_3_ slabs on different dielectrics, focusing the light beam along material boundaries into a single spot by considering the polariton’s incident angle and boundary orientation. Furthermore, they demonstrated negative reflection of HPhPs [46], enabling polaritons with different momenta to reflect to a common focal point, utilizing a concept similar to their previous study [45] in the design of a hyperbolic nanoresonator. Diverging from the antenna shaping technique [42], [43], [44], [45], [46], Hu et al. [47] have proposed an approach involving the partial coverage of half of the *α*-MoO_3_ slab with a monolayer graphene to accomplish planar tunable focusing. HPhPs generated by metallic antenna shaping converge at a focal point through wavelet interference at the edge, altering the incident light upon coupling with the metallic disk. In contrast, negative refraction modifies the propagation path of pre-excited light to achieve focusing. This approach allows focusing of pre-excited divergent waves, *in situ* manipulation of focusing, and dynamic control of focusing through electrical gating of graphene’s *E*_*f*_.

In this study, we introduce an innovative approach using the isofrequency contours (IFCs) in wavevector space for optimizing interface geometry by patterning a graphene layer to a graphene disk. The divergent wavefronts are induced and propagate toward the edges of the graphene. These graphene edges are crucial in launching in-phase wavefronts that align with the hyperbolic wavefronts along the [100] direction, as supported by the bare *α*-MoO_3_ slab. This alignment facilitates the convergence of hyperbolic wavefronts at focal points, making the proposed approach significantly augments the focusing qualities encompassing focal length, lateral dimensions, power intensity, and beam profile. Additionally, the selective coverage of an additional graphene sheet can unlock a fixed focal length. Our research presents a foundation for developing a considerably efficient and versatile focusing system, with potential applications in IR optical imaging, chemical and biosensing, and photonic waveguiding.

## 2 Patterned graphene on an *α*-phase molybdenum trioxide slab

**Figure 1:**
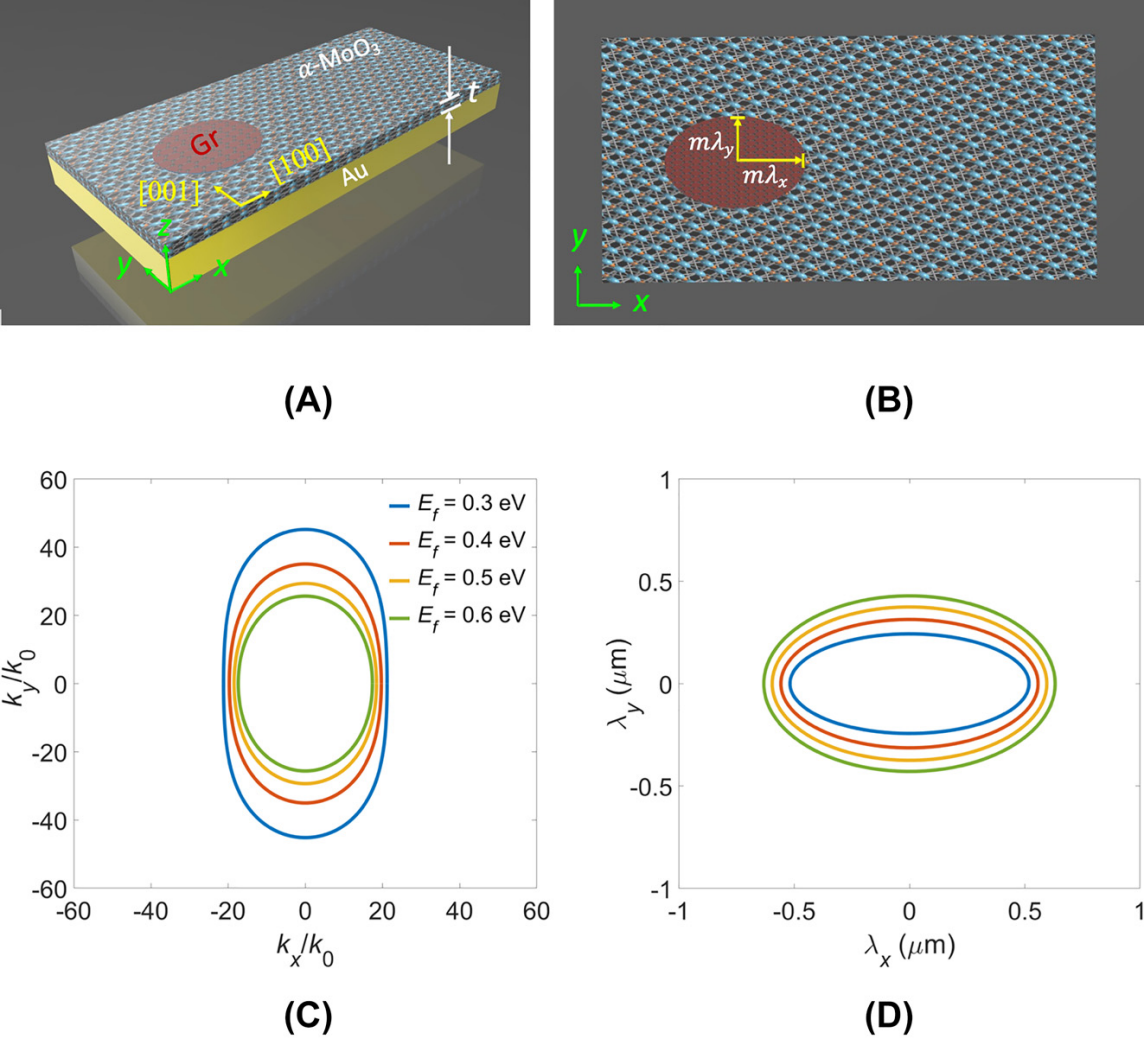
The proposed structure and its top view. (A) Illustration of the current structure, comprising a patterned graphene (Gr) layer on an *α*-MoO_3_ slab with thickness *t* situated on an Au substrate. The *z*-polarized electric dipole source is denoted as *E*_*z*_, whereas the principal axes of the *α*-MoO_3_ crystal are identified as [100] and [001]. (B) Top view of the structure, where *m* signifies a positive real number, and *λ*_*x*_ and *λ*_*y*_ represent the polariton wavelengths along the *x* and *y*-directions, respectively. The isofrequency contours (IFCs) and the associated geometric profiles of graphene. (C) Analytical IFCs for the air/graphene/*α*-MoO_3_/Au system at various *E*_*f*_ values at a fixed *t* of 150 nm for the *α*-MoO_3_ slab. (D) The accompanying geometric profiles of graphene in the scales of *λ*_*x*_ and *λ*_*y*_.

*α*-MoO_3_, a natural biaxial vdW crystal, exhibits in-plane hyperbolic dispersion and highly anisotropic permittivity, enabling the propagation of HPhPs in mid-IR Reststrahlen bands (RBs). We focused on RB II, spanning a frequency range of 816–972 cm^−1^. In this context, crystallographic directions [100], [001], and [010] of *α*-MoO_3_ corresponded to coordinates *x*, *y*, and *z*, respectively, revealing the permittivity characteristics of *ε*_*x*_ < 0, *ε*_*y*_ > 0, and *ε*_*z*_ > 0. In Figure 1A, we depict the structure under consideration, consisting of a patterned graphene (indicated in red ellipse), an *α*-MoO_3_ slab with thickness *t*, and an underlying Au substrate, arranged from top to bottom, respectively. The crystallographic direction [100] of *α*-MoO_3_ aligned with the *x*-direction, unless specified otherwise. In particular, a gold (Au) substrate results in significantly stronger field compression and lower normalized propagation loss for the polaritons compared to those on a low-loss dielectric substrate [40]. This is due to the coupling of the polaritonic mode of an *α*-MoO_3_ slab with its mirror image in the metal, forming a new 2D mode – the image polariton [28], [29]. This mode is equivalent to the symmetric second order in a double-thickness *α*-MoO_3_ slab. As such, the Au substrate plays a crucial role in considerably compressing the spot size of the focus in this study. Figure 1B provides a top view of the structure, where *m* represents a multiple of polariton wavelengths and can be a positive real number. *λ*_*x*_ and *λ*_*y*_, whose calculation will be explained in the subsequent section, represent the analytical polariton wavelengths along the *x* and *y*-directions, respectively.

Before investigating the characteristics of the proposed structure, the fabrication steps for the proposed structure are briefly outlined as follows: (1) High-quality *α*-MoO_3_ flakes are prepared through mechanical exfoliation of bulk crystals synthesized via chemical vapor deposition (CVD); (2) The *α*-MoO_3_ flakes are transferred onto gold substrates using a dry transfer process with a polydimethylsiloxane stamp; (3) A graphene monolayer is grown on copper foil and subsequently transferred onto the *α*-MoO_3_ flakes using a poly(methyl methacrylate)-assisted method [48]; (4) The graphene sheet is patterned into ellipses using electron beam lithography (EBL) [49], [50] or through direct synthesis via CVD [51], [52]; In direct synthesis, Narita et al. [51] successfully fabricated closely packed graphene nanodisks with diameters of 60 nm and a 30 nm spacing using EBL; Katzmarek et al. [52] fabricated graphene grating widths of at least 100 nm. Given that the dimensions of the patterned structure in this study are on the order of micrometers, the fabrication of our patterned graphene is feasible. Experimentally, the nanotip of s-SNOM initiates polaritons across all positions while scanning the sample surface. The scanning outcomes are subsequently correlated with the field distributions of Re(*E*_*z*_). Experimentally, an automated dual-tip s-SNOM using tip launching approach has recently been reported [53], [54], [55], but the presence of an avoidance area, formed during the scan of a detection tip around the excitation tip, restricts access to the complete near-field pattern. Therefore, employing a far-field excitation scheme through the placement of an Au nanoantenna on the graphene ellipse, where Au antennas can be patterned using EBL followed by a lift-off process [56] on the patterned graphene surface as optical antennas, illuminated by a *p*-polarized incident plane wave, remains the current feasible and popular approach [40], [47].

To emulate the nanoimaging of polaritons captured by s-SNOM, we conducted tip launching approach to model the near-field distribution by introducing a *z*-polarized electric dipole positioned 50 nm above the center of the graphene ellipse in COMSOL with a computational domain enclosed under a perfect-matched-layer boundary condition to minimize undesirable reflections, ensuring the accuracy of the results. The electric field distribution is then probed at 20 nm above the surface of the *α*-MoO_3_ flakes. The analytical permittivity of the *α*-MoO_3_ was determined using the Lorentz model [34], [47] (see Supplementary Material, §1.1). The graphene sheet was modeled as an infinitesimally thin layer with surface current density, and its surface conductivity was computed using the Kubo formula [57] (see Supplementary Material, §1.2). The complex permittivity of Au was found in the literature [58]. Upon obtaining the profiles of *E*_*z*_ fields, the corresponding IFCs in the wavevector space (*k*_*x*_, *k*_*y*_) were computed via spatial Fourier transformation, with the sampling resolutions set at *N*_*x*_ = 1000 and *N*_*y*_ = 1000.

To tailor the geometric patterns of graphene, we first analyzed a four-layer slab waveguide comprising air/graphene/*α*-MoO_3_/Au [47]. This analysis enabled us to obtain analytical IFCs at various Fermi levels (*E*_*f*_), as depicted in Figure 1C. To ensure wavefront matching at the boundaries of the patterned graphene, we transformed the IFCs from the wavevector space to spatial domains. The spatial scaling was determined based on the polariton wavelength, denoted as *λ*_*i*_ = *λ*_0_ *k*_0_/*k*_*i*_, as shown in Figure 1D. Here, *i* denotes a specific direction within the *x*–*y* plane. Notably, the geometrical profile of the patterned graphene resembles that of a prolate ellipse, characterized by long and short axes represented by *λ*_*x*_ and *λ*_*y*_ along the *x* and *y*-directions, respectively. It can be observed that the extent of the prolate ellipse increased with *E*_*f*_ due to a looser energy confinement. Moreover, the focal length, *f* can be flexibly adjusted by multiplying *λ*_*x*_ and *λ*_*y*_ by a factor of *m*.

## 3 Results and discussions

### 3.1 Focusing performances of polaritons

**Figure 2:**
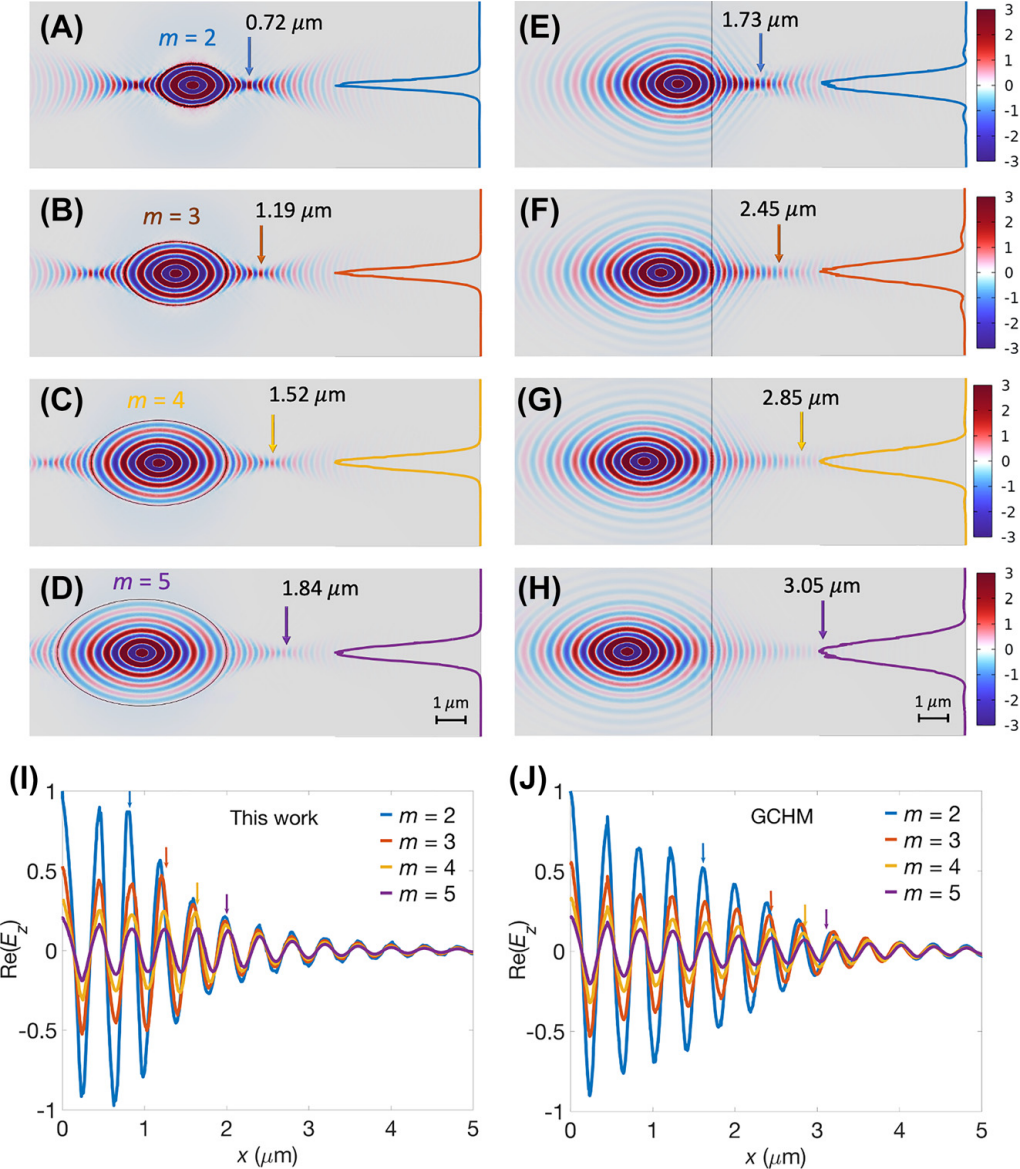
Re(*E*_*z*_) field distributions for the proposed structure at various “*m*” values, including (A) *m* = 2, (B) 3, (C) 4, and (D) 5. The corresponding Re(*E*_*z*_) field distributions for the structure in reference [47] are illustrated for (E) *m* = 2, (F) 3, (G) 4, and (H) 5. These distributions are obtained using consistent parameters, including *ω* = 910 cm^−1^, *α*-MoO_3_ slab *t* of 150 nm, and *E*_*f*_ of 0.5 eV. Normalized Re(*E*_*z*_) line plots are provided on the right-hand side along the *y*-direction of the focal length, *f*. (I) Normalized Re(*E*_*z*_) line fields for the proposed structure are depicted along the *x*-direction, starting from the graphene edge (*x* = 0) to the position of *x* = 5 μm. (J) Normalized Re(*E*_*z*_) line plots start from the interface to the position of *x* = 5 μm for the structure in Ref. [47]. The focal points for different *m* values are indicated by arrows.

To demonstrate the focusing characteristics of the proposed structure, we determined the spatial distributions of the real part of electric field component in the *z*-direction Re(*E*_*z*_) and the corresponding values of *f* for *m* ranging from 2 to 5, as shown in Figure 2A–D, respectively. The value of *m* is measured from the excitation source to the graphene–MoO_3_ interface (rightmost edge of graphene ellipse) of the GCHM (proposed) structure. These simulations were conducted under specific parameters: angular frequency (*ω*) = 910 cm^−1^, *t* = 150 nm, and *E*_*f*_ = 0.5 eV. Further, we included the results from a previous study [47], which adopted a plain graphene sheet covering half of an *α*-MoO_3_ slab (GCHM), as shown in Figure 2E–H. The vertical dashed lines in Figure 2A–D mark the right edges of graphene ellipses, and the distance between these edges and the dipole source is *mλ*_*x*_. Similarly, the vertical solid lines in Figure 2E–H indicate the interfaces between the left graphene-covered and right bare *α*-MoO_3_ slabs, with the distance between the interface and the dipole source also equal to *mλ*_*x*_. It was observed that exciting the polaritons on the patterned graphene initiates their propagation outward in various directions. Upon reaching the edges of the graphene ellipse, these polaritons undergo topological shifts from elliptical to hyperbolic IFCs. Consequently, their spreading becomes confined along the [100] direction, transitioning into the hyperbolic wavefront supported by the bare *α*-MoO_3_ slab, thereby enhancing light focusing. Contrarily, the GCHM required a relatively long distance to converge the divergent wavefront to the focal point because a relatively broad wavefront was required at the interface.

Polaritons propagate within damping media; thus, a high *f* results in high energy loss. A stronger power intensity at the focal point enhances imaging quality and light–matter interaction. It is noteworthy that a smaller *m* correlates with a shorter *f*, as shown in Figure 2A–D, leading to a stronger power intensity due to propagation over a shorter distance in lossy media. Therefore, reducing the *f* is advantageous for preserving energy, improving imaging quality, and enhancing light–matter interaction. The proposed structure exhibited *f* values of 0.72 μm at *m* = 2 and 1.84 μm at *m* = 5, representing only 0.42 and 0.60 times the respective values of the GCHM. Remarkably, with increasing *m* towards infinity, the proposed structure gradually approaches the behavior of the GCHM, indicating that the GCHM was a special case of the proposed structure. Due to the need for a relatively broad wavefront at the interface, the GCHM necessitates an extended distance to converge the divergent wavefront to the focal point. Furthermore, the Re(*E*_*z*_) distributions at the values of *f* along the *y*-direction exhibited significant ripples near the main lobe for the GCHM, as depicted on the right side of Figure 2E–H. These ripples considerably impacted the focal quality, reducing its overall performance. In comparing the field profiles for various *m* values, we presented the normalized Re(*E*_*z*_) line plots of the proposed structure along the *x*-direction. These fields started at the graphene edge and extended to the position of *x* = 5 μm, as depicted in Figure 2I. Similarly, we illustrated the corresponding normalized Re(*E*_*z*_) line plots for the GCHM, which started at the interface and spanned to the position of *x* = 5 μm, as shown in Figure 2J. The arrows within these figures indicate the focal points for different *m* values. Notably, the field profiles shown in Figure 2I and J are normalized by the field amplitudes of *m* = 2 in their respective structures. In our proposed structure, the field amplitudes at the focal points were at their maximum values (see Figure 2I). However, the field amplitudes at the focal points were not the maximum (see Figure 2J) in the GCHM. This was because the energy loss due to the high *f* in the GCHM exceeded the energy enhancement achieved through the focusing process. In particular, we observed that the distances between the dipole source and the graphene edge coincidentally aligned with the values of *f*, revealing a mirror imaging effect.

**Figure 3:**
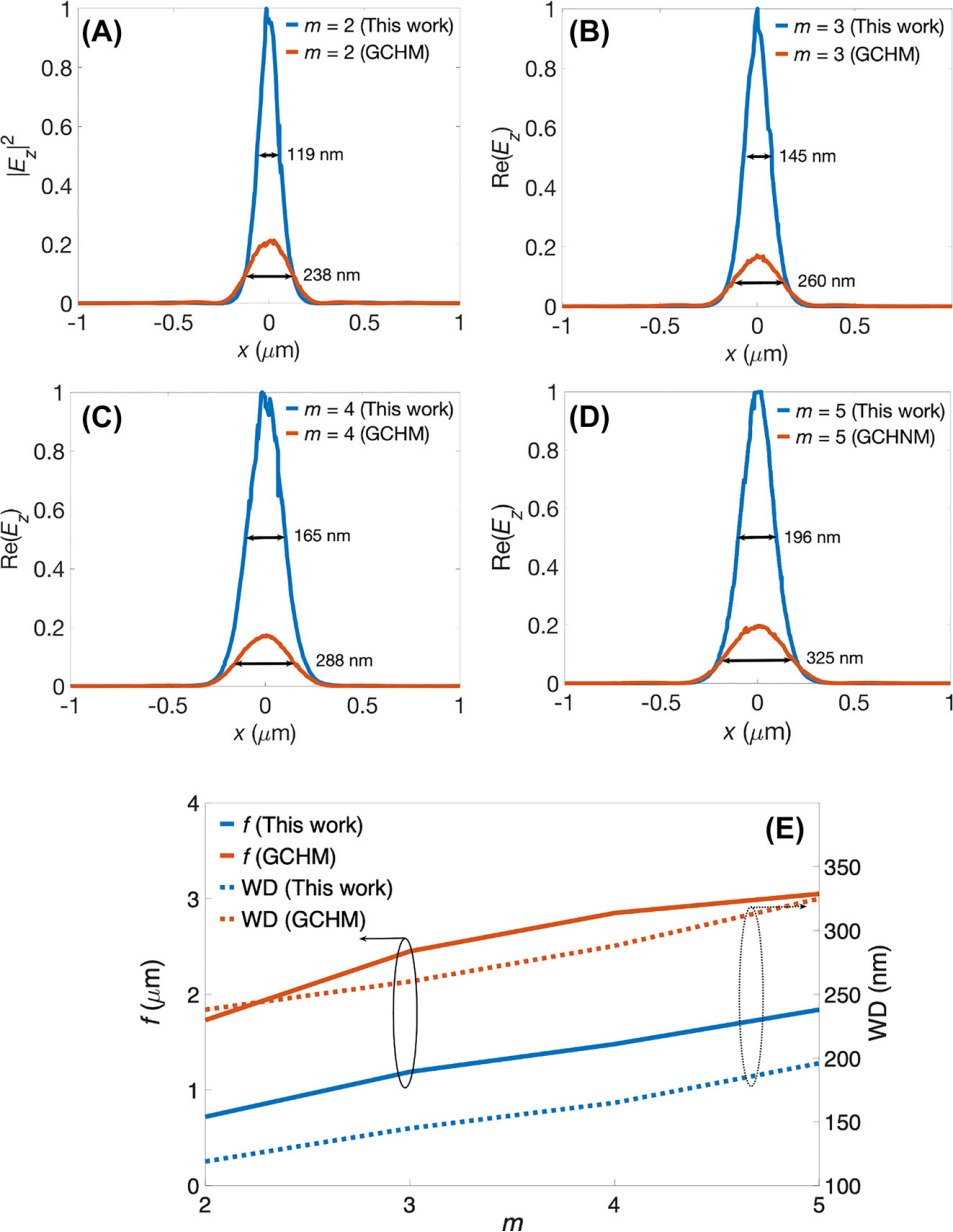
Profiles of the |*E*_z_|^2^, values of *f*, and waist diameters (WDs). Field intensities of the |*E*_*z*_|^2^ of the proposed structure and GCHM for (A) *m* = 2, (B) 3, (C) 4, and (D) 5 and the associated WDs, where the |*E*_*z*_|^2^ line plots are normalized by those of the proposed structure. (E) *f* and WD are depicted as functions of *m* for both structures.

To investigate the wavevector characteristics of our proposed structure, we conducted a Fourier transformation of the Re(*E*_*z*_) field profiles presented in Figure 2A–D. The resulting numerical IFC plots in the wavevector space (*k*_*x*_, *k*_*y*_) are depicted in Figure S1A–D for *m* = 2–4, respectively, overlaid with the corresponding analytical IFCs. The IFCs for the Re(*E*_*z*_) fields within the graphene ellipse and the bare *α*-MoO_3_ exhibited elliptical and hyperbolic profiles, respectively. Notably, the thickness of the brightest ellipse in the IFC diminished as *m* increased. This reduction was attributed to the small spatial extent of the graphene ellipse for low *m* values, resulting in a thick IFC in the wavevector domain. Additionally, the number of ellipses between the center and the brightest ellipse corresponded to the value of *m*, as explained by the presence of the same number of polariton wavelengths within the graphene ellipse. To quantitatively assess the waist diameter (WD) and light intensity, we plotted the line intensities of |*E*_*z*_|^2^ at the focal points for the proposed structure and GCHM. These plots are displayed in Figure 3A–D for different values of *m* ranging from 2 to 5, respectively. Remarkably, our observations revealed that the intensities in the proposed structure were approximately five times stronger than those in the GCHM. Additionally, the WD in our design achieved approximately 60 % of the values in the GCHM. For *m* = 2 (5), the WD corresponded to approximately 1/92 (1/54) or equivalently 1.09 % (1.85 %) of the free-space light wavelength of 10.99 μm (*ω* = 910 cm^−1^). To observe the focusing characteristics, we plotted the *f* and WD as functions of *m*, as shown in Figure 3E, for both the proposed structure and GCHM. It showed that the *f* and WD exhibited linear dependencies on *m*, indicating that large graphene ellipses resulted in high values of *f* and WDs. These results indicated that the proposed design significantly enhanced focusing qualities. These included achieving a lower *f*, cleaner beam profile, higher power intensity, and lower WD than the previously published results in the GCHM. The realization of an ultrasmall spot in the proposed structure enabled deep subwavelength focusing, coupled with strong light–matter interaction for mid-IR polaritons.

### 3.2 Focusing characteristics’ dependence on Fermi level, operating frequency, and noninteger positive real number

**Figure 4:**
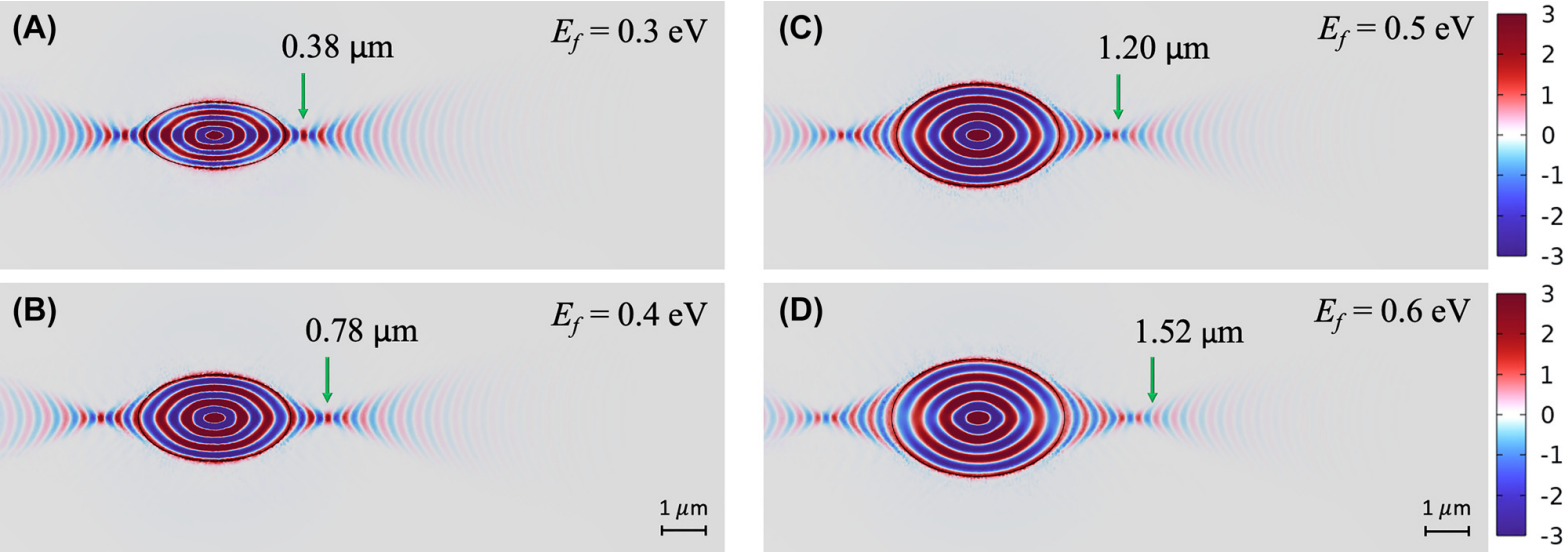
Re(*E*_*z*_) field distributions of the proposed structure for *E*_*f*_ = (A) 0.3, (B) 0.4, (C) 0.5, (D) 0.6 under the parameters of angular frequency *ω* = 910 cm^−1^, *t* = 150 nm, and *m* = 3.

We delved deeper into the dependencies of the focusing characteristics of our proposed structure on varying *E*_*f*_. The Re(*E*_*z*_) distributions, corresponding to different values of *E*_*f*_ ranging from 0.3 to 0.6 eV, are shown in Figure 4A–D under the conditions of *ω* = 910 cm^−1^, *t* = 150 nm, and *m* = 3. Note that the polariton wavelength increased as *E*_*f*_ increased, thereby increasing the geometric size of the graphene ellipse. We observed that the *f* increased by approximately one polariton wavelength (*λ*_*pb*_ = 0.40 μm) in the bare *α*-MoO_3_ slab for every additional increment of 0.1 eV within the range of *E*_*f*_ from 0.3 to 0.6 eV. These results are consistent with the notion that a high *m* affords a high *f*. The *f* can be actively controlled in a range of 0.38–1.52 μm when varying *E*_*f*_ from 0.3 to 0.6 eV, respectively. Notably, the WDs remained at approximately 135 nm, with minimal variations as *E*_*f*_ changed.

Considering the impact of the spectral response on focusing characteristics, we examined the Re(*E*_*z*_) fields across the frequency range from *ω* = 880 to 930 cm^−1^ under the condition of *E*_*f*_ = 0.5 eV, *t* = 150 nm, and *m* = 3. The results are shown in Figure S2A–F. The polariton wavelength on the graphene ellipse increased as *ω* decreased, affording a large graphene ellipse. Additionally, the polariton wavelength increased, supported within the bare *α*-MoO_3_ slab, as *ω* decreased, further augmenting the *f*. Consequently, the variation in the *f* while altering *ω* was more pronounced than the changes observed while varying the *E*_*f*_. The *f* varied from 4.01 to 0.43 μm and WD varied from 176 to 116 nm as *ω* was adjusted across the range from 880 to 930 cm^−1^, illustrating the significant influence of spectral variation on the focusing characteristics. The Re(*E*_*z*_) fields at *m* = 3–3.9, with increments of 0.1, are exhibited in Figure S3A–J, respectively. The field profiles within the graphene ellipses exhibited slightly different interference patterns when noninteger values of *m* were employed. This confirmed that the values of *f* could be subtly adjusted by increasing *m* in a noninteger manner, without the constraint of integer values.

### 3.3 Diffractionless propagation and directional steering

**Figure 5:**
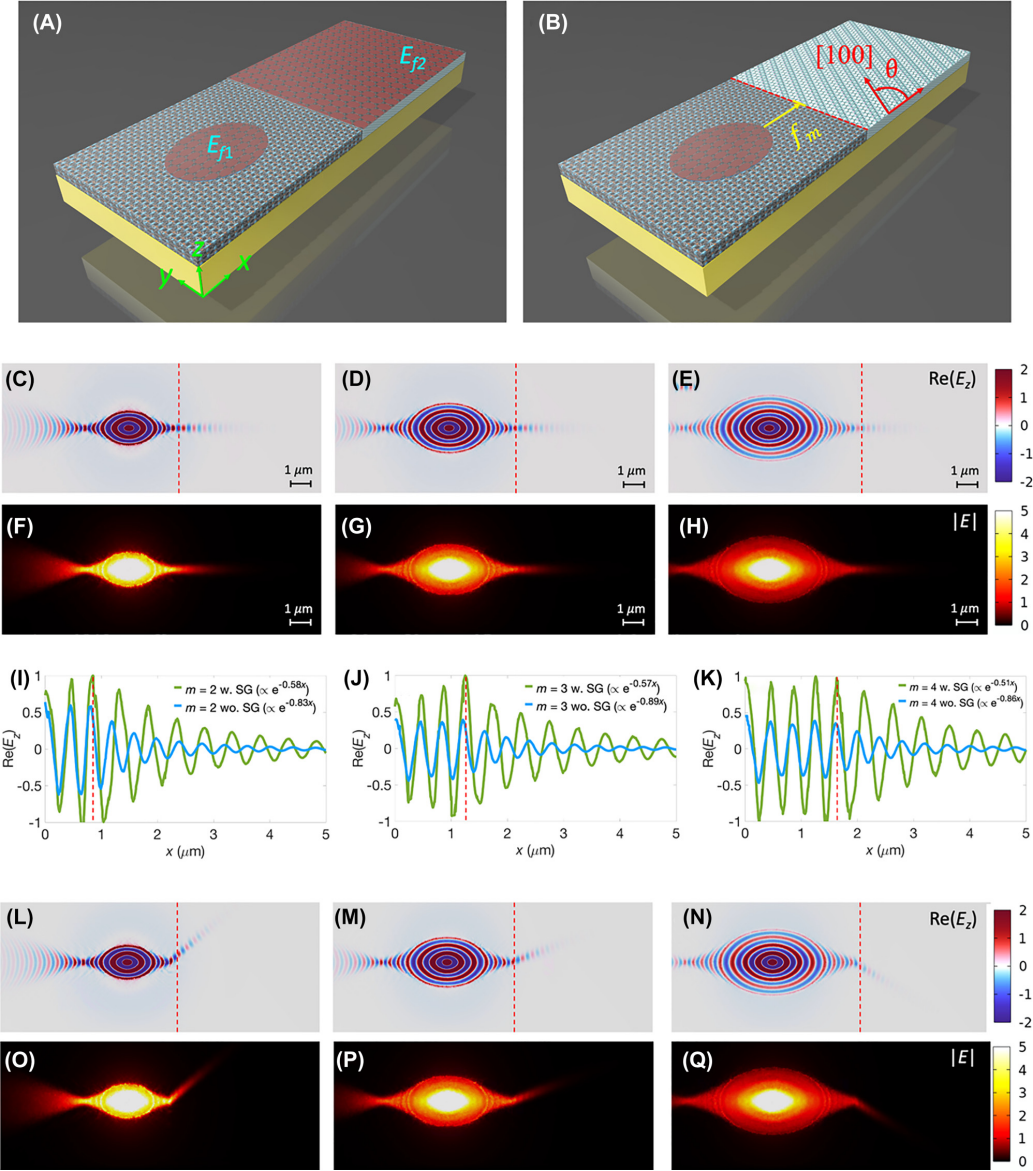
Achieving diffractionless propagation and directional steering. (A) A composite structure featuring two graphene patterns: an elliptical ellipse with *E*_*f*1_ and a semi-infinite graphene (SG) sheet with *E*_*f*2_ placed on two differently oriented *α*-MoO_3_ slabs. (B) The structure shown in part (A) without the SG, revealing the oriented *α*-MoO_3_ slab at an angle *θ*, with *f*_*m*_ denoting the *f* for a specific *m*. Profiles of Re(*E*_*z*_) at *m* = (C) 2, (D) 3, and (E) 4 and |*E*| at *m* = (F) 2, (G) 3, and (H) 4. These profiles are obtained under the following parameters: *ω* = 910 cm^−1^, *t* = 150 nm, *E*_*f*1_ = 0.5 eV, and *E*_*f*2_ = 0.22 eV. Notably, the red dashed lines highlight the interfaces between the two differently oriented *α*-MoO_3_ slabs. Line plots of Re(*E*_*z*_) along the *x*-direction with and without the SG for *m* = (I) 2, (J) 3, and (K) 4. Re(*E*_*z*_) profiles with SG under the conditions of (L) *m* = 2 and *θ* = 40°, (M) *m* = 3 and *θ* = 20°, and (N) *m* = 4 and *θ* = −30°. Corresponding |*E*_*z*_| profiles are shown under the conditions (O)–(Q).

To further steer the focusing light beam, we employed a composite structure that combined two differently oriented *α*-MoO_3_ slabs, each covered with distinct graphene patterns, as depicted in Figure 5A. The left portion of this composite structure is identical to that shown in Figure 1A. However, the right side features an oriented *α*-MoO_3_ slab at an angle *θ* between the *x*-axis and the crystallographic direction [100] of the *α*-MoO_3_, as illustrated in Figure 5B, and is covered with a semi-infinite graphene (SG) sheet. The permittivity tensor of the *α*-MoO_3_ slab with an oriented angle *θ* could be obtained by conducting coordinate transformation with a rotation matrix (see Supplementary Material, §4). The graphene patterns were electrically gated with distinct values of *E*_*f*_, denoted as *E*_*f*1_ for the left ellipse and *E*_*f*2_ for the right sheet. The separation between the edge of the ellipse and the interface of the two *α*-MoO_3_ slabs was set as the *f*, indicated as *f*_*m*_ for different values of *m*. To fabricate a lateral heterojunction combined with two differently oriented *α*-MoO_3_ flakes as shown in Figure 5A or B, step (2) would be replaced by the interfacial sliding approach proposed by Li et al. [59]. Initially, a vertical heterostructure is prepared on a polyimide (PI) substrate by stacking two *α*-MoO_3_ flakes vertically. Then, by applying tensile strain to the flexible PI substrate via mechanical bending, the *α*-MoO_3_ flakes can gradually slide apart or toward each other during the substrate stretching process. By controlling the tensile strain, the two contacted flakes will eventually separate and convert into two distinct flakes. In our previous work [60], the focusing performances under a plain graphene sheet covering the left *α*-MoO_3_ slab, which had no orientation to the *x*-axis, were identified as needing substantial improvement, particularly due to significant ripples near the main lobe impacting the focal quality. As illustrated in Figure 5A of [60], a gap was set between both sides of the graphene sheet without graphene coverage, requiring precise tuning of its width. In contrast, in the current design, the left boundary of the right graphene sheet aligns directly with the focal point, facilitating perfect light canalizations. This is made possible because the patterned graphene achieves high-quality beam profiles and narrower waist diameters at the focal points, surpassing those obtained with the plain graphene sheet.

The specific parameters employed for these simulations were as follows: *ω* = 910 cm^−1^, *t* = 150 nm, *E*_*f*1_ = 0.5 eV, *E*_*f*2_ = 0.22 eV, *θ* = 0°, and various values of *f*_*m*_ (*f*_2_ = 0.72 μm, *f*_3_ = 1.19 μm, and *f*_4_ = 1.52 μm). Re(*E*_*z*_) and |*E*| profiles are shown in Figure 5C–E and F–H, respectively, ranging from *m* = 2 to 4. Notably, the red dashed lines in these figures denote the interfaces between the two *α*-MoO_3_ slabs. The WDs were well preserved as the focusing beams traversed the interfaces, thereby canalizing the polaritons and unlocking the fixed values of *f*. This contrasted the conventional diffraction exhibited by polaritons without the SG, as depicted in Figure 2A–C. Furthermore, to evaluate the field enhancement achieved by adding SG, the Re(*E*_*z*_) fields are depicted in Figure 5I–K for values of *m* = 2–4, respectively. These results revealed a significant reduction in the decay rate of field amplitude, transitioning from approximately *e* ^−0.89*x*^ for the structure without SG to *e* ^−0.57*x*^ for the structure with SG at *m* = 3. This outcome suggests that the proposed approach offers a versatile imaging method that does not impose restrictions based on fixed values of *f*. Furthermore, we steered the direction of the focused polaritons by adjusting the angle *θ* of the right *α*-MoO_3_ slab. The Re(*E*_*z*_) profiles for three conditions, (1) *m* = 2 and *θ* = 40°, (2) *m* = 3 and *θ* = 20°, and (3) *m* = 4 and *θ* = −30°, are presented in Figure 5L–N, respectively, and their associated |*E*_*z*_| profiles are illustrated in Figure 5O–Q. These results provide compelling evidence to effectively control the directional canalization of polaritons. The outcomes presented herein demonstrate that our proposed approach enables high-quality focusing and facilitates the directional waveguiding of mid-IR polaritons. This establishes a viable system for constructing a diverse range of photonic circuits and imaging systems.

## 4 Conclusions

The theoretical analyses and numerical simulations are conducted to demonstrate high-quality and tunable mid-IR light focusing utilizing a 2D vdW material, *α*-MoO_3_, adorned with patterned graphene. Our proposed approach enabled direct control over the propagation path of excited light, exploiting the phenomenon of negative refraction. When compared with the graphene interface, our patterned graphene exhibited superior focusing characteristics: (1) remarkably low waist diameter of 119 nm, merely 1/92 of the incident wavelength in free-space, (2) exceptional beam profile quality, and (3) a notable fivefold increase in field intensity. Additionally, the spot size of the focused beam was effectively maintained, ensuring its propagation in a canalized manner, thereby preventing conventional beam diffraction. This was achieved by introducing a graphene layer positioned beyond the focal points. Furthermore, our approach enabled the directional steering of the focused beam to the desired direction by twisting the graphene-covered *α*-MoO_3_ slab. The present approach provides an additional degree of freedom for manipulating the mid-IR light propagation characteristics.

## Supplementary Material

Supplementary Material Details
