# Supplementary material for: Focusing of mid-infrared polaritons through patterned graphene on van der Waals crystals
Source: Nanophotonics. 2024 Apr 15;13(15):2753–63. doi: 10.1515/nanoph-2023-0778 (PMC11501983; doi:10.1515/nanoph-2023-0778)
Supplement: Supplementary file 1 — Supplementary Material Details [file j_nanoph-2023-0778_suppl_001.docx]

**Supplementary Material**

Ruey-Tarng Liu, Yan-Ze Wu, and Chia-Chien Huang^^[[1]](#endnote-1)^*^

**Focusing and steering of mid-infrared polaritons through patterned graphene on van der Waals crystals**

1. **Material parameters of *α*-phase molybdenum trioxide and graphene**

**1.1 Permittivity of** ***α*-phase molybdenum trioxide**

The permittivity of *α*-phase molybdenum trioxide (*α*-MoO_3_) was calculated by the following Lorentz model:


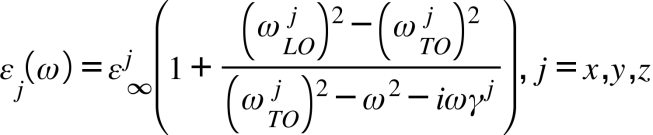
, (S1)

where 𝜀*_j_* denotes the principal component of the permittivity tensor;
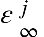
(
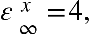

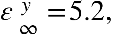

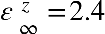
)denotes the high-frequency permittivity in the *j* direction;
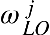
(cm^-1^) (
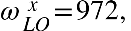

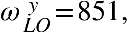

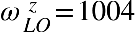
) and (
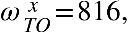

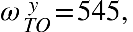

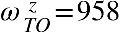
) denote the longitudinal optical and transverse optical phonon frequencies, respectively;
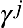
(
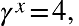

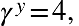

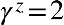
) denotes the inelastic loss rate.

**1.2 Surface conductivity of graphene**

The surface conductivity of graphene, denoted as *σ*, was computed using the Kubo formula. It comprised both intraband and interband contributions and can be expressed as follows:


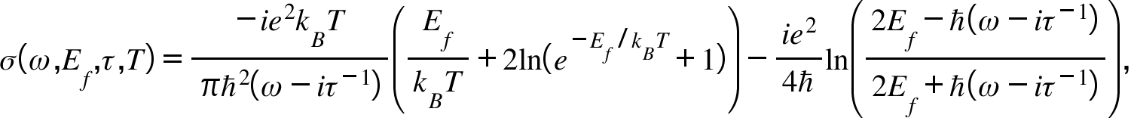
 (S2)

where the parameters include the angular frequency (*ω*), Fermi level (*E_f_*), carrier relaxation lifetime (*τ*) = *µE_f_* /*ev_f_*^2^, absolute temperature (T), electron charge (*e*), Boltzmann constant (*k_B_*), reduced Planck constant (ℏ), carrier mobility (*µ*), and Fermi velocity of electrons (*v_f_*) = 10^6^ m/s. To avoid damage to the ellipse graphene and minimize the influence of the near-field signal when employing electrical gating, the proper approach to tuning the *E_f_* of the graphene discs is by applying conventional chemical doping in the proposed system. In this investigation, we assumed a practical value of *µ* = 1,000 cm²/V·s at a room temperature of *T* = 300 K.

1. **Isofrequency contours for Re(*E_z_*) fields**

We conducted a Fourier transformation of the Re(*E_z_*) field profiles, as illustrated in Figure 2A–D, affording numerical isofrequency contour (IFC) plots in wavevector space (*k_x_*, *k_y_*) corresponding to different values of *m*. To enhance the clarity of the numerical solutions, we plotted only the left half of the analytical IFCs of the hyperbolic profiles.


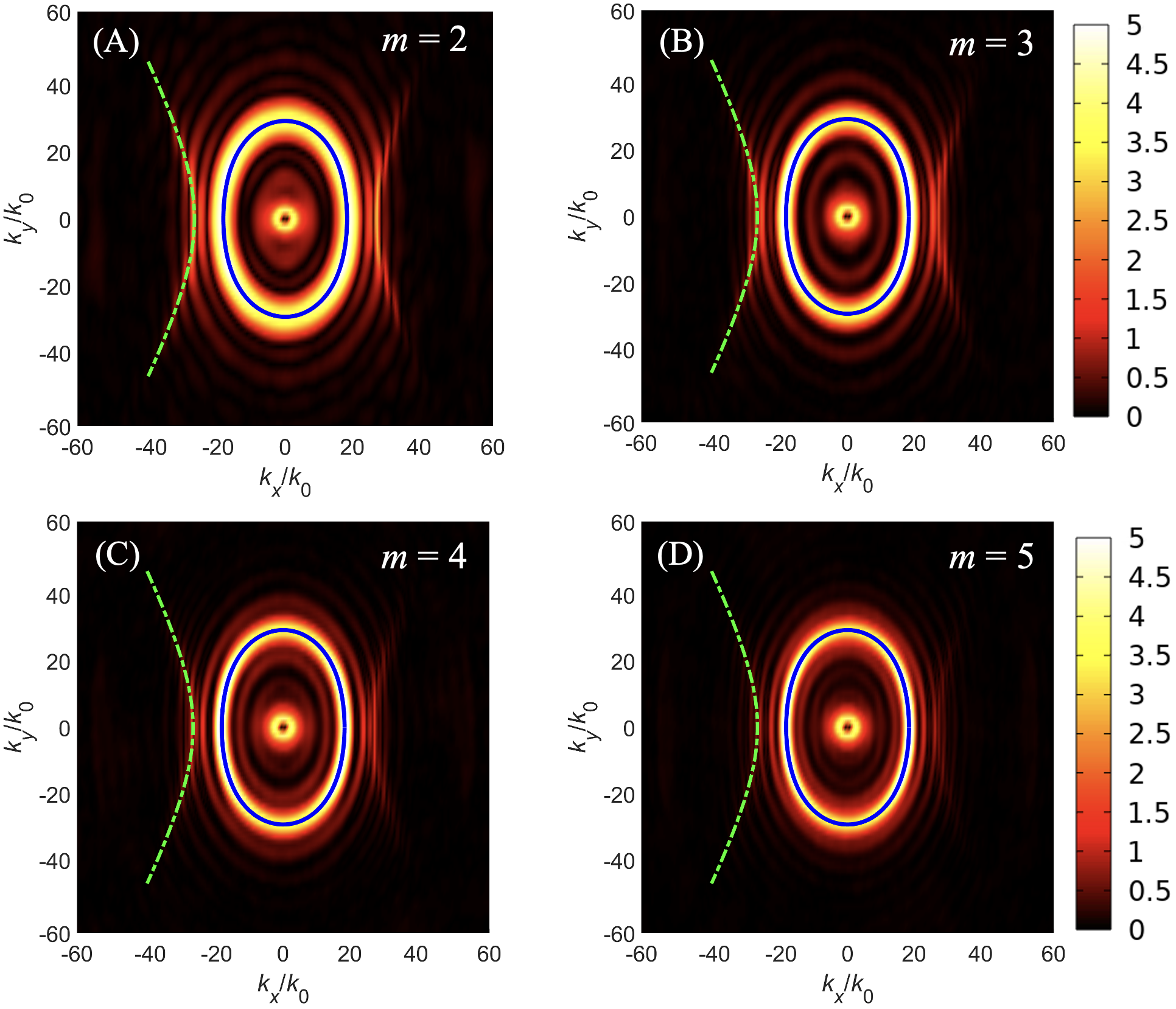


**Figure S1:** Isofrequency contours (IFCs) for Re(*E_z_*) fields corresponding to (A) *m* (a positive real number) = 2, (B) 3, (C) 4, and (D) 5 under the conditions of angular frequency (*ω*) = 910 cm^−1^, thickness (*t*) = 150 nm, and Fermi level (*E_f_*) = 0.5 eV. Analytical IFCs are represented by the green dashed and cyan solid lines for reference.

1. **Focusing characteristics**

**3.1 Operating frequency**


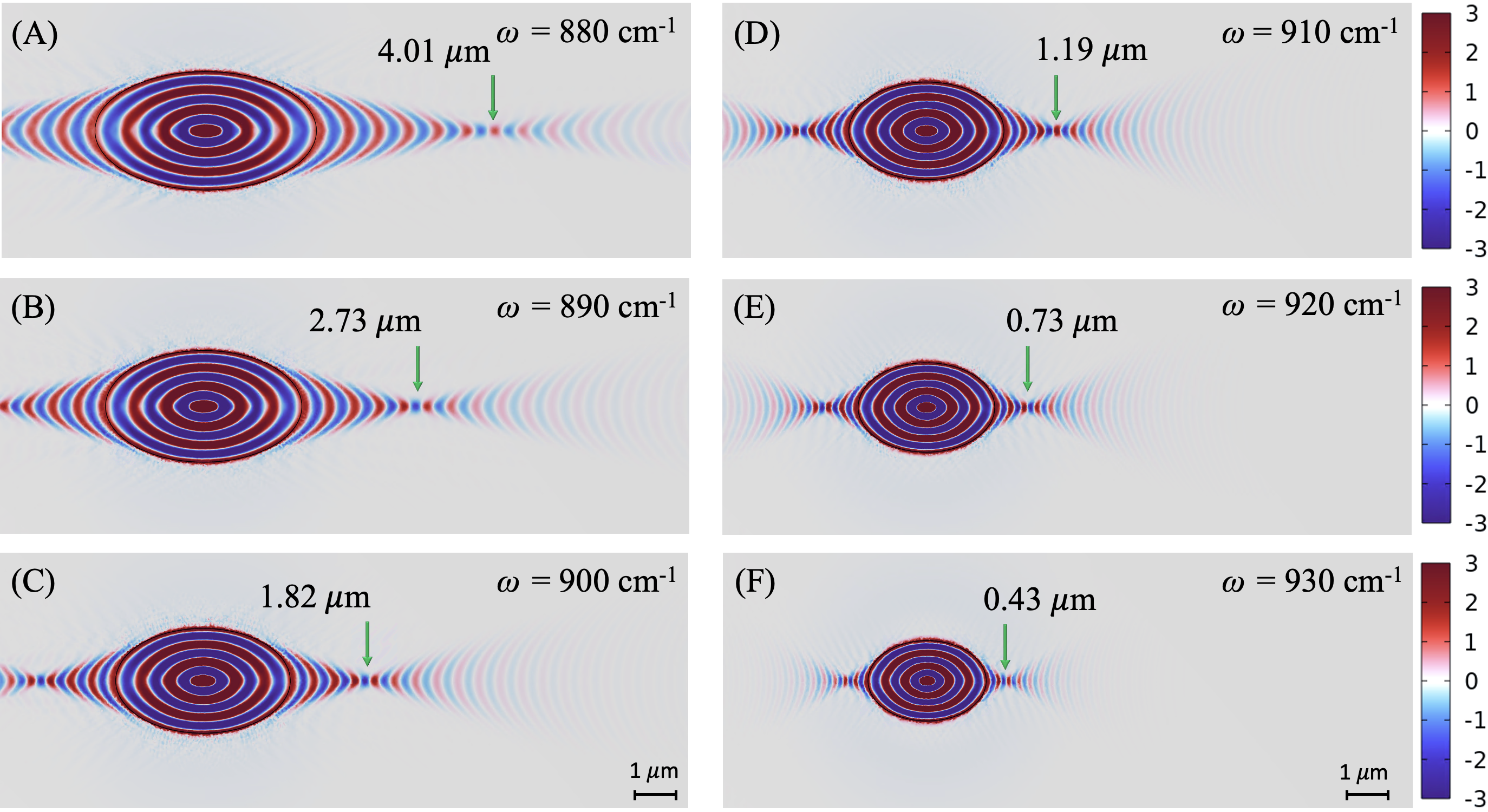


**Figure S2:** The Re(*E_z_*) fields of the proposed structure for *ω* = (A) 880, (B) 890, (C) 900, (D) 910, (E) 920, and (F) 930 cm^−1^ under the parameters of *E_f_* = 0.5 eV, *t* = 150 nm, and *m* = 3.

**3.2 Noninteger positive real number**


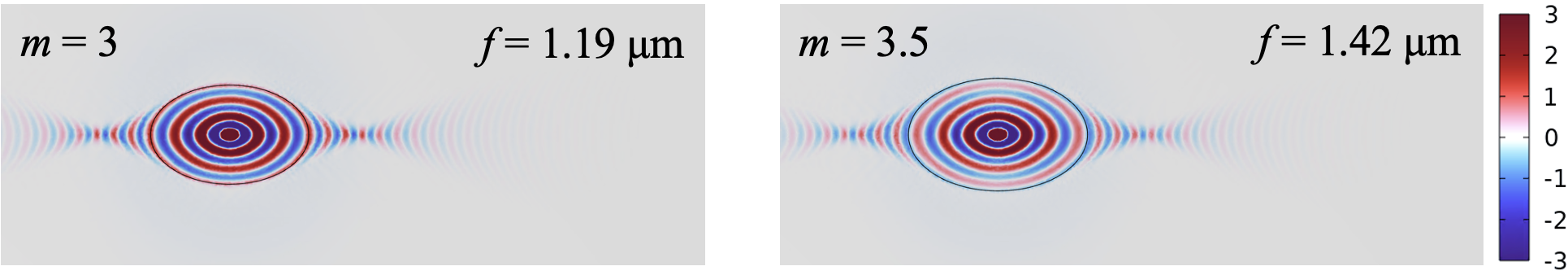


(A) (F)


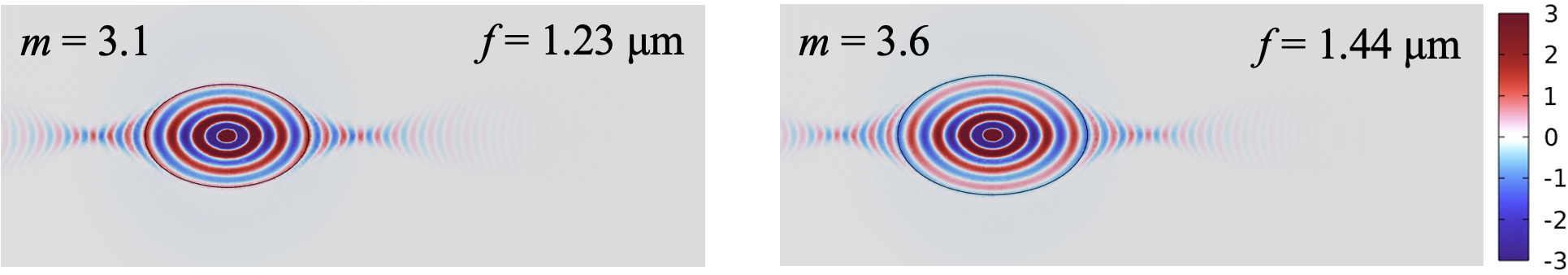


(B) (G)


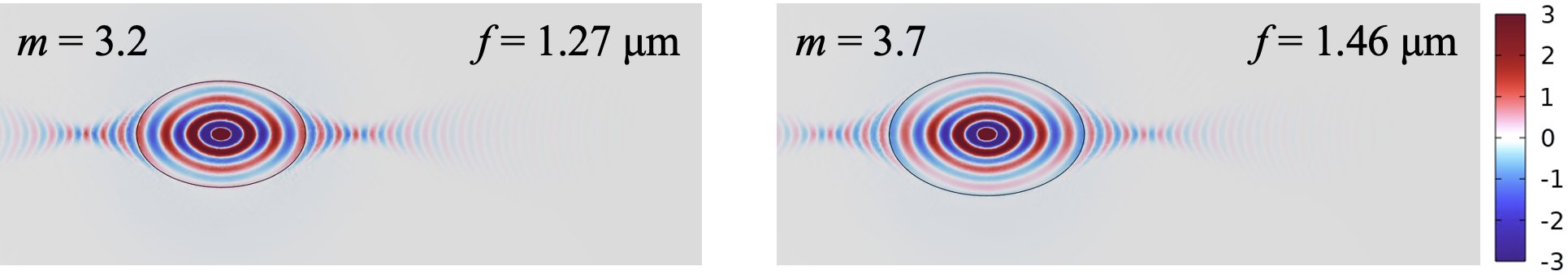


(C) (H)


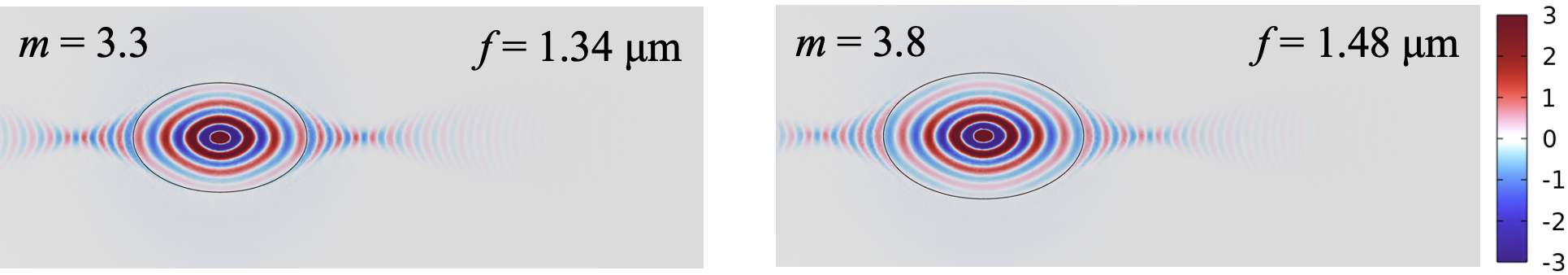


(D) (I)


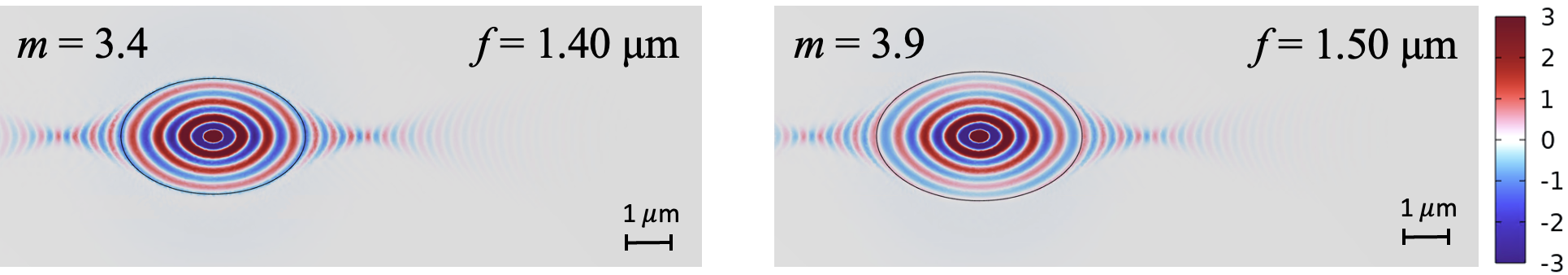


(E) (J)

**Figure S3:** Re(*E_z_*) fields for *m* = (A) 3, (B) 3.1, (C) 3.2, (D) 3.3, (E) 3.4, (F) 3.5, (G) 3.6, (H) 3.7, (I) 3.8, and (J) 3.9 with the corresponding focal lengths at the parameters of *ω* = 910 cm^−1^, *E_f_* = 0.5 eV, and *t* = 150 nm.

1. **Permittivity of *α*-phase molybdenum trioxide with an orientation of angle *θ***

For an *α*-MoO_3_ slab with the crystallographic direction [100] aligning with the *x*-direction, the permittivity tensor is expressed as ***ε*** *=* diag[*ε_x_*, *ε_y_*, *ε_z_*], where *ε_x_*, *ε_y_*, and *ε_z_* are the permittivity components along the principal axes [100], [001], and [010], respectively, of the *α*-MoO_3_ slab. By rotating an *α*-MoO_3_ slab counterclockwise around the *z*-axis, altering the angle *θ*, which corresponds to the orientation of the principal axis [100] for the *x*-axis and *θ* is positive for counterclockwise rotation from the *x*-axis, the oriented permittivity tensor is expressed as ***ε****^θ^ =****RεR****^T^*, where the rotation matrix (***R***) and ***ε****^θ^* are as follows:


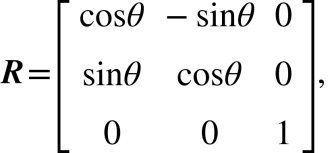
 (S3)


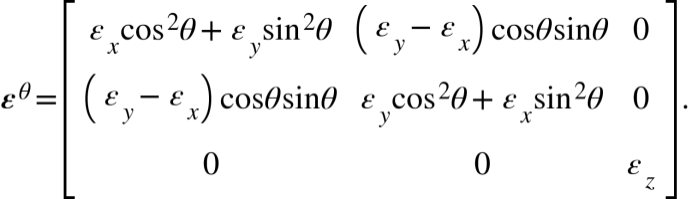
 (S4)

1. [↑](#endnote-ref-1)
